# Optimization of extracranial stereotactic radiation therapy of small lung lesions using accurate dose calculation algorithms

**DOI:** 10.1186/1748-717X-1-45

**Published:** 2006-11-29

**Authors:** Barbara Dobler, Cornelia Walter, Antje Knopf, Daniella Fabri, Rainer Loeschel, Martin Polednik, Frank Schneider, Frederik Wenz, Frank Lohr

**Affiliations:** 1 Department of Radiation Oncology, Mannheim Medical Center, University of Heidelberg, Mannheim, Germany; 2 Department of Radiotherapy, Regensburg University Medical Center, Regensburg, Germany; 3 Department of Computer Science and Mathematics, University of Applied Sciences, Regensburg, Germany

## Abstract

**Background:**

The aim of this study was to compare and to validate different dose calculation algorithms for the use in radiation therapy of small lung lesions and to optimize the treatment planning using accurate dose calculation algorithms.

**Methods:**

A 9-field conformal treatment plan was generated on an inhomogeneous phantom with lung mimics and a soft tissue equivalent insert, mimicking a lung tumor. The dose distribution was calculated with the Pencil Beam and Collapsed Cone algorithms implemented in Masterplan (Nucletron) and the Monte Carlo system XVMC and validated using Gafchromic EBT films. Differences in dose distribution were evaluated. The plans were then optimized by adding segments to the outer shell of the target in order to increase the dose near the interface to the lung.

**Results:**

The Pencil Beam algorithm overestimated the dose by up to 15% compared to the measurements. Collapsed Cone and Monte Carlo predicted the dose more accurately with a maximum difference of -8% and -3% respectively compared to the film. Plan optimization by adding small segments to the peripheral parts of the target, creating a 2-step fluence modulation, allowed to increase target coverage and homogeneity as compared to the uncorrected 9 field plan.

**Conclusion:**

The use of forward 2-step fluence modulation in radiotherapy of small lung lesions allows the improvement of tumor coverage and dose homogeneity as compared to non-modulated treatment plans and may thus help to increase the local tumor control probability. While the Collapsed Cone algorithm is closer to measurements than the Pencil Beam algorithm, both algorithms are limited at tissue/lung interfaces, leaving Monte-Carlo the most accurate algorithm for dose prediction.

## Background

In external beam radiation therapy of lung tumors, even in extracranial stereotactic high dose ablative treatments, in-field relapses are observed even at high doses [1-3].

It is well known that the Pencil Beam dose calculation algorithm (PB), which is most commonly implemented in commercial treatment planning systems, has limited accuracy especially at the interface of tissues with large differences in electron density, as it is the case at the interface lung/tumor [4]. Collapsed Cone convolution methods (CC) and Monte Carlo simulations (MC) are able to predict the dose distribution with a higher accuracy [5-10]. They are, however, more time consuming and therefore implemented in a few commercial treatment planning systems only and not widely used in clinical routine yet.

Several studies have been published, which report the differences in dose distributions in inhomogeneous media predicted by the different dose calculation algorithms. Martens et al [8] reported an overestimation of the dose in the upper-airway mucosa by the Pencil Beam algorithm. Haedinger et al [10] and Koelbl et al [9] found that the Pencil Beam algorithm overestimates the dose to targets in the lung as compared to the Collapsed Cone algorithm, which may lead to an insufficient dose to the target volume. These reports, however, do not include any 2D dose measurements in anthropomorphic phantoms or investigations about how the treatment plans can be improved in order to achieve the prescribed dose at the periphery of the target without increasing lung toxicity.

The aim of this study was to optimize the treatment of small lung tumors with regard to target coverage and dose homogeneity using accurate dose calculation algorithms and to validate the results in an anthropomorphic phantom. The first step was to quantify the error introduced when creating plans using the Pencil Beam dose calculation algorithm by recalculating plans with Collapsed Cone, Monte Carlo and comparing them to absolute dose measurements in an inhomogeneous thoracic phantom. Once the error was quantified, the plans were optimized by adding small segments to the peripheral parts of the target, creating a 2-step fluence modulation. Similar to what is done in inverse planning of IMRT, this fluence modulation is used to create a steeper dose gradient at the interface of the target to the lung, in order to increase the dose to the periphery of the target while keeping the dose to the lung low. To achieve a homogeneous dose distribution for dose escalation in a larger volume of the target, the weights of the segments were altered to determine the optimal weights for the desired dose homogeneity using Collapsed Cone and Monte Carlo calculations. The calculations were validated by 2D film dosimetry.

## Methods

### Phantom Design

**Figure 1:**
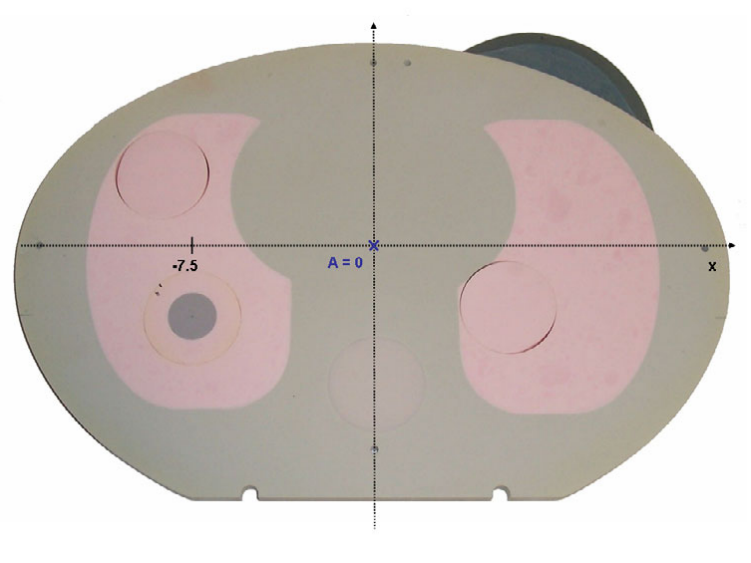
Slice of the inhomogeneous phantom with mimics for normal tissue, lung tissue, bone and lung tumor.

An anthropomorphic phantom based on the inhomogeneous thoracic phantom 002LFC of CIRS Tissue Simulation Technology (Norfolk, Virginia, USA) was designed as shown in fig 1. The phantom is made of a 15 cm block and 15 1 cm slices of tissue equivalent material with different densities for lung, bone, normal tissue, and tumor tissue. The size of the phantom is 30 cm × 20 cm × 30 cm. The electron densities of the material relative to water are 1.003 for normal tissue, 0.207 for lung tissue and 1.506 for bony tissue. For the CT number to electron density conversion, additional plugs are available with relative electron densities of 1.042 for muscle and 0.949 for adipose tissue. Rods for ionization chambers can be inserted in the different tissue parts of the phantom and films can be placed in between the slices for 2D dosimetry. To mimic coin lesions in the lung, tissue equivalent plugs of 1 cm to 3 cm diameter and normal tissue density were manufactured which can be inserted in the lung tissue of the phantom. In this study a tumor plug of 2 cm diameter and 4 cm height was used.

### Dose Calculation Algorithms

Dose calculations were performed using the Pencil Beam algorithm and the Collapsed Cone algorithm implemented in Masterplan (Nucletron BV, Venendal, the Netherlands). The algorithms are described in detail by Ahnesjö et al [11,12]. For the Monte Carlo simulations XVMC including the Elekta Synergy treatment head model was kindly supplied by Matthias Fippel [13,14]. A detailed overview over the different dose calculation methods has been given by Ahnesjö and Aspradakis [15].

### Treatment Plans

**Figure 2:**
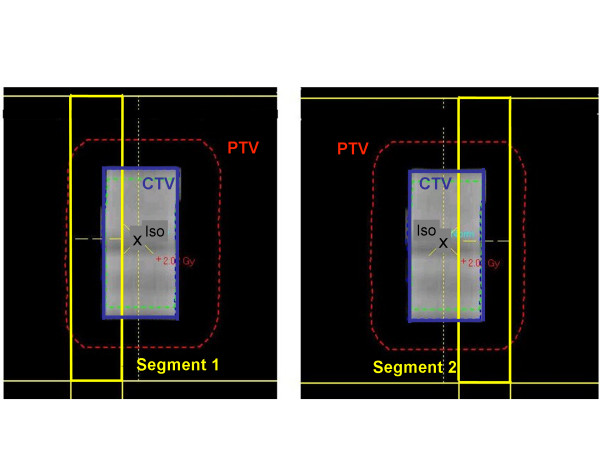
Beam's Eye View of the supplementary peripheral segments of a beam.

For the study a CT scan of the phantom described above with a tumor insert of 2 cm diameter was acquired (slice thickness 3 mm) and transferred to the treatment planning systems. A 9 field coplanar treatment plan was created in Masterplan for the Elekta Synergy Linac (Elekta, Crawley, UK), 6MV photon energy, with gantry angles of 195°, 255°, 285°, 315°, 345°, 15°, 45°, 140°, and 165° and rectangular symmetric fields of 4.8 cm width and 7.2 cm height. The plan was calculated with the Pencil Beam algorithm implemented in Masterplan with a prescription dose of 2Gy to the isocenter. Then the plan was recalculated with the Collapsed Cone algorithm implemented in Masterplan and the XVMC Monte Carlo system using the number of monitor units that resulted from the Pencil Beam calculation with a sum over all beams of 245 MU. The fraction dose of 2Gy to the isocenter was chosen for dosimetry reasons. In clinical practice a fraction dose of 10Gy to 20Gy, depending on the fractionation regimen, would have been applied. This, however, is beyond the range of the films. For optimization, two additional asymmetric segments of 1.3 cm width and 7.2 cm height each were created for each beam, covering the peripheral parts and excluding the central part of the target (fig 2) in order to achieve the desired dose at the periphery of the target. Plans were calculated with different weighting of the monitor units of the small segments relative to the monitor units of the open field ranging from 1/4 to 1/10 as well as one plan with zero monitor units for selected segments (asymmetric approach). For small numbers of monitor units, the numbers were rounded in the calculation to fit the rounding of MU at the linac. The calculations were initially performed with the faster Collapsed Cone algorithm and then recalculated by Monte Carlo simulations.

### Dosimetric Validation

Dosimetric validation was performed by absolute film dosimetry using the Gafchromic^® ^EBT radiochromic film (ISP, New Jersey, USA) and the EPSON 1680 scanner [16-20]. The properties of the film in combination with the EPSON 1680 scanner were thoroughly investigated prior to the use in this study. The overall error in absolute dose is highly dependent on the film handling and scanner settings and can be as much as 40% if inappropriate settings are used. Sources of error are e.g. the use of an automatic color correction, the increase of scanner temperature and exceeding exposure of the film to light. The use of proper scanner settings and proper film handling, however, allow to reduce the maximum error to 3% [21] in the central region (10 cm × 20 cm) of the scanner. In the periphery of the scanning field, i.e. more than 10 cm from the center, the error can be as much as 8% without applying further corrections due to the horizontal inhomogeneity of the scanner [22].

The setup error of the phantom at the linac was estimated as ± 1 mm. The influence of the setup error on the measured dose distribution in cranio-caudal direction was eliminated by using a phantom geometry which does not change in the cranio-caudal direction (cylindrical targets). Fore spherical targets, even small setup errors in the cranio-caudal direction cause a change in target diameter in the transversal plane. This would have added an additional uncertainty in the evaluation which could be eliminated by using cylindrical targets.

### Moving Targets

To test target coverage for moving targets, moving targets were simulated by shifting the isocenter relative to the phantom by 5 mm in the lateral direction and recalculating the plan. Even though the main direction of target movement is in the cranio-caudal direction, the lateral direction was used because there is no fluence modulation in cranio-caudal direction and lateral movement of our cylindrical insert also mimics cranio-caudal movement of a clinical lesion that is typically spherical.

### Evaluation

The evaluation of the accuracy of dose calculation was performed by comparison of dose profiles and difference matrices of the calculated and measured doses. Evaluation of plan optimization was performed by means of Dose Volumes Histograms (DVHs) calculated by CC and PB and comparison of dose profiles of the calculated and measured dose matrices. DVHs of MC calculated plans are not available since our version of XVMC does not support dose volume histograms.

## Results

### Comparison of dose calculation algorithms for the 9 field plan

**Figure 3:**
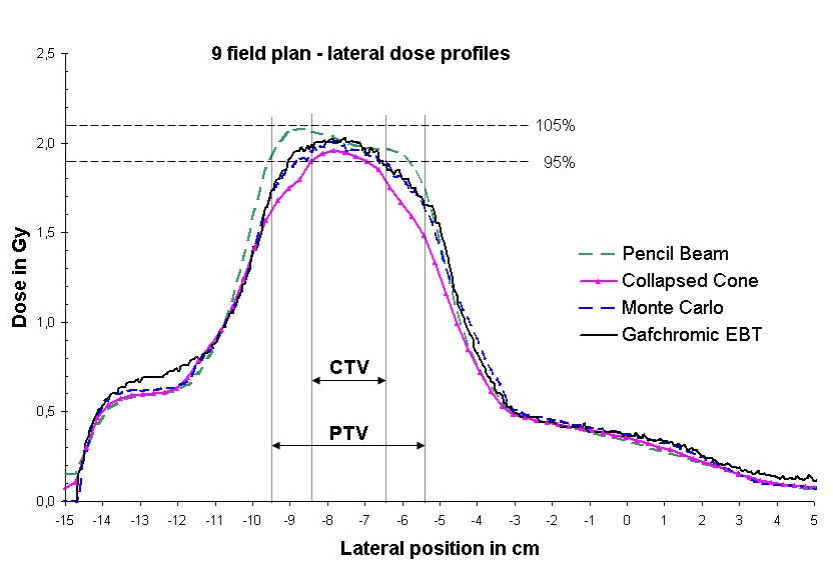
Lateral dose profiles for the 9 field plan calculated with Pencil Beam, Collapsed Cone, and Monte Carlo and measured by film dosimetry. Overestimation of the dose by PB by up to 15%, underestimation by CC by up to -8% and by MC by up to -3% as compared to the measurement.

The dose distributions of the 9 field treatment plan calculated with Pencil Beam, Collapsed Cone and Monte Carlo were compared to each other as well as to the film measurements in the phantom. Lateral dose profiles through the isocenter are given in fig 3. Evaluation was performed separately for the CTV, i.e. the tumor tissue equivalent volume of the phantom, and the PTV, which extended the CTV by 1 cm into the lung equivalent tissue of the phantom. Inside the CTV, the Pencil Beam algorithm overestimated the dose by up to 5.4% compared to the measurements, while the Collapsed Cone algorithm underestimated the dose by up to 5.0%. The maximum errors were found at the interface tumor tissue to lung tissue in both cases. For Monte Carlo the dose difference inside the CTV compared to the measurements was below 2% with a maximum error of -1.9%. For the PTV, the maximum differences between dose calculation and measurement were found in the lung tissue part of the phantom: There, the dose was overestimated by the Pencil Beam algorithm by 15% and underestimated by the Collapsed Cone algorithm by 8%. For the Monte Carlo calculations the maximum dose difference compared to the measurements was found to be 3%.

**Figure 4:**
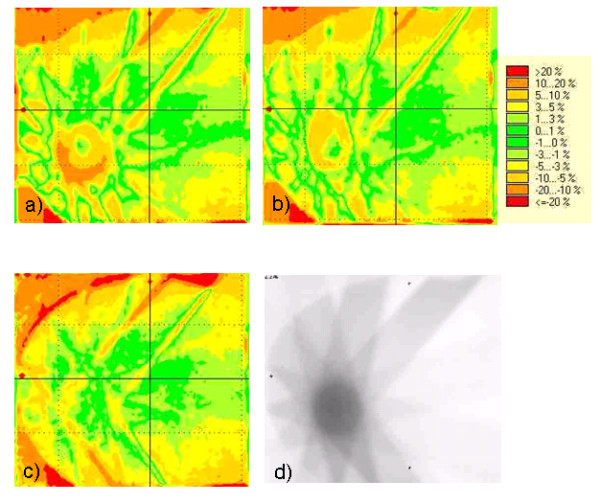
Difference Matrices of measured – calculated dose for the different dose calculation algorithms in the isocenter slice for the 9 field plan: a) Pencil Beam b) Collapsed Cone c) Monte Carlo d) Gafchromic EBT film associated with the difference matrices. The agreement of calculated with measured dose improves from PB to CC to MC.

Figure 4 shows 2D dose difference matrices in the isocenter plane for the different dose calculation methods compared to the film measurement. The highest deviations between calculated and measured dose in a large part of the high dose area, i.e. the PTV, can be found for the PB (10%–20%). CC is more accurate but still with a 5%–10% deviation in the major part (ca. 75%) of the PTV. For MC deviations are less than 3% in the major part of the PTV. In some very small spots of the high gradient area, deviations in the 10%–20% region can be found also for CC and MC. These were, however, caused by the limited accuracy of the alignment of film and calculated dose matrix in the verification software.

### Plan optimization

**Figure 5:**
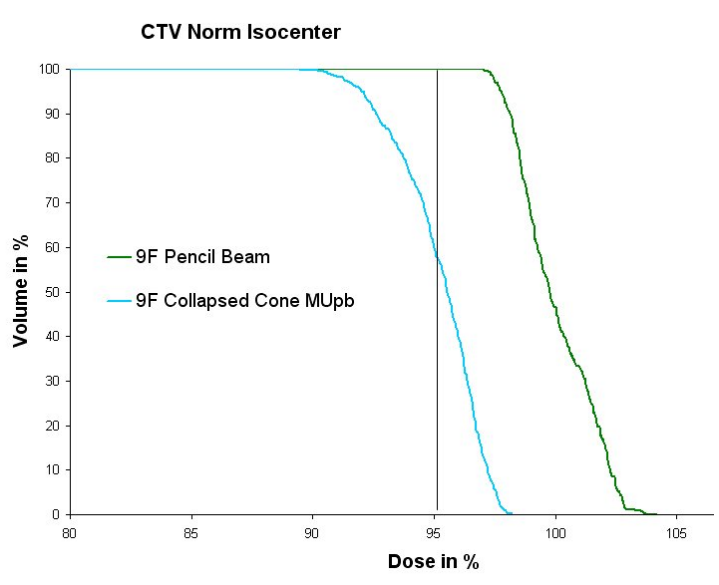
Comparison of DVHs for the 9 field plan as calculated with Pencil Beam and Collapsed Cone for the same number of Monitor Units.  Normalization was performed to the isocenter for Pencil Beam. The volume of the CTV covered by the 95% isodose which is predicted by PC to be 100%, is only 61% if calculated by CC.

The DVHs calculated by PB and CC for the same number of Monitor Units (MU) are given in fig 5. The dose was normalized to the isocenter in the plan calculated with PB. Comparison of the DVHs shows that the volume of the CTV covered by the 95% isodose which is predicted by the PB to be 100%, is only 61% if calculated by CC.

**Figure 6:**
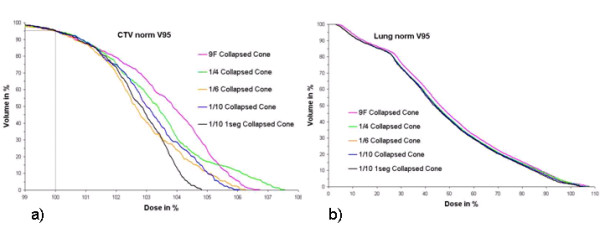
Comparison of DVHs for the optimized plans, calculated with Collapsed Cone: a) CTV b) right lung. Normalization was performed to the dose encompassing 95% of the volume for better visualization. The highest dose homogeneity is achieved by the 1/10 weighting for one peripheral segment per beam (black line), while the dose to the lung is even slightly lower than for the 9 field plan.

Plan optimization by additional peripheral segments of different weights resulted in the DVHs given in fig 6. Normalization was performed to the dose covering 95% of the CTV in this case in order to increase target coverage as compared to the MU calculated by PB. The peripheral segments of different weights are used to optimize dose homogeneity. As can be derived from fig 6, the best homogeneity was achieved for a weighting of the peripheral segments of 1/10 of the large segments and only one peripheral segment per beam. The dose to the lung was slightly lower for this plan than for the 9 field plan. In turn, this also means that by adding extra segments minimum dose to the tumor could be increased while keeping lung exposure almost unchanged. Lower weighting of the peripheral segments was not possible for a prescription dose of 2Gy because the linac handles only integer MU. Since only 6 slices of the phantom of 1 cm each were used for the dose calculation, the total volume of the lung was much smaller than in a real patient. Therefore the DVHs of the lung can only be used for phantom plan comparison but not for comparisons to real patient plans.

**Figure 7:**
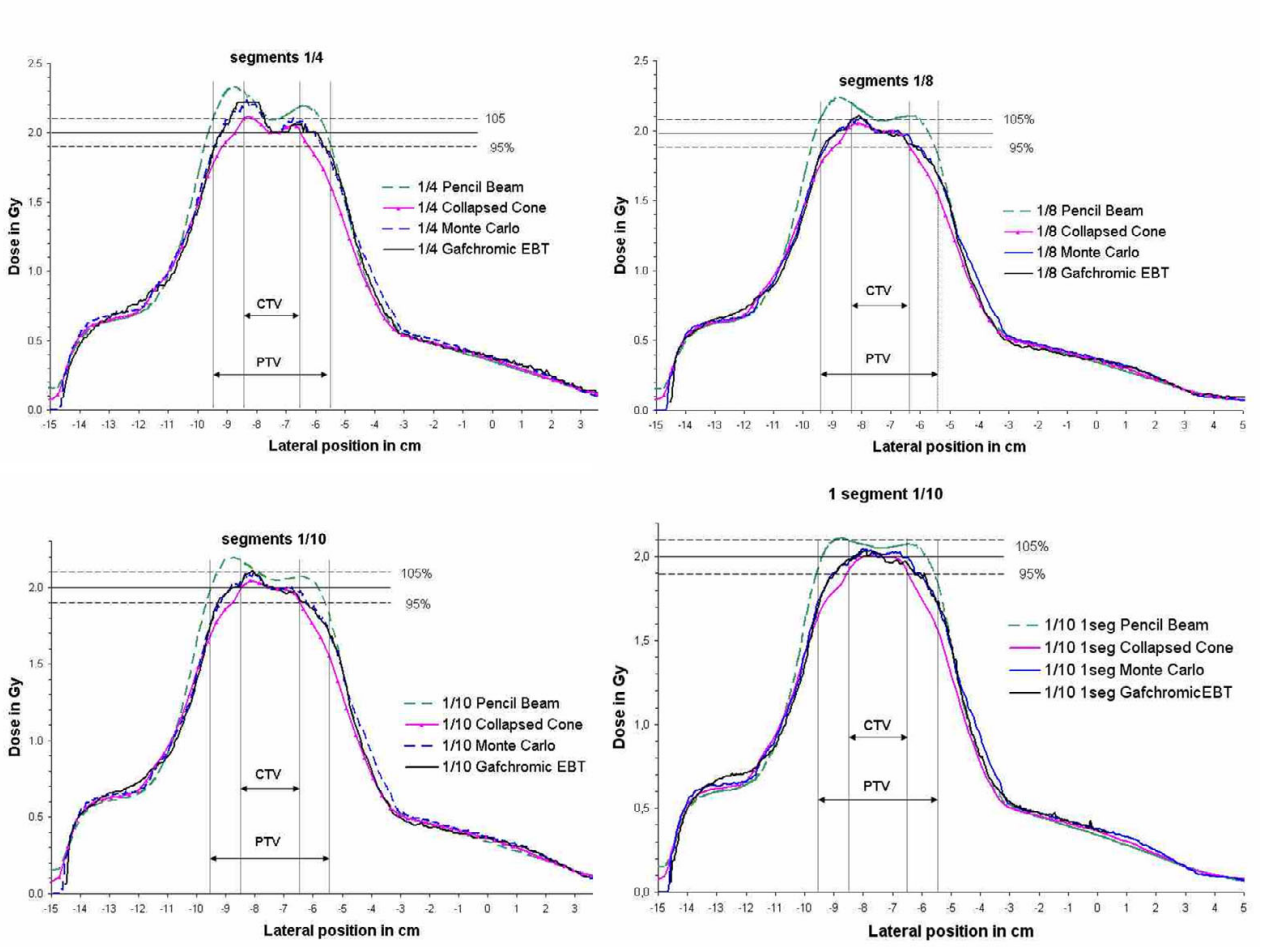
Lateral dose profiles for the optimized plans as calculated by Pencil Beam, Collapsed Cone, and Monte Carlo and measured by film dosimetry. Normalization was performed to the isocenter for the Collapsed Cone. Weighting of the peripheral segments compared to the large segment: a) 1/4 b) 1/8 c) 1/10 d) only one peripheral segment per beam with weight 1/10. The comparison validates the increasing accuracy from PB to CC to MC. MC calculation and film measurement confirm that the highest homogeneity is achieved for the 1/10 weighting of one peripheral segment per beam.

Figure 7 shows dose profiles for the plans with different weightings of the small peripheral segments, calculated with Pencil Beam, Collapsed Cone and Monte Carlo, and validated by absolute film dosimetry. Comparison of calculated versus measured dose confirmed the overestimation of the dose by the Pencil Beam, the underestimation by the Collapsed Cone, and the agreement of the film with the Monte Carlo calculations. Despite the remaining inaccuracy of the Collapsed Cone algorithm, the result of the optimization process with Collapsed Cone was confirmed by the Monte Carlo calculations as well as the film measurements.

### Moving targets

**Figure 8:**
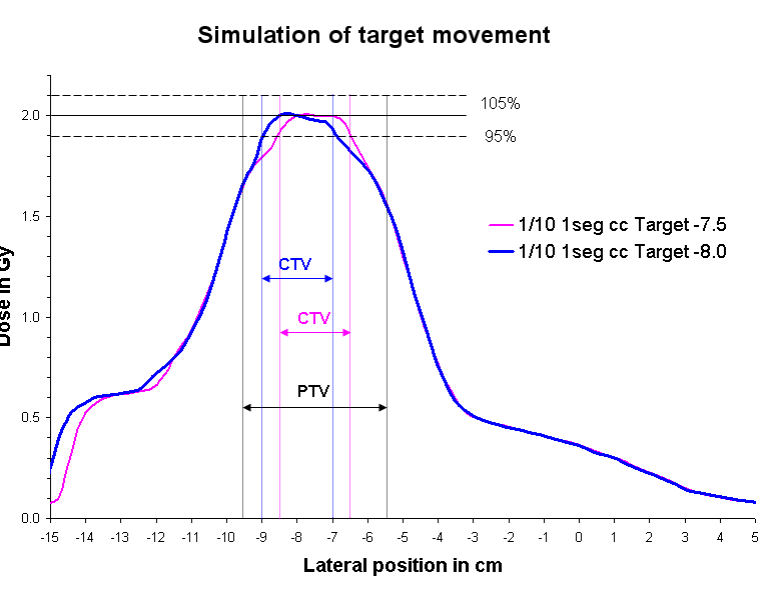
Moving targets were simulated by shifting the isocenter relative to the phantom by 5mm in the lateral direction and recalculating the plan with the Collapsed Cone algorithm. The original CTV (center at -7.5cm) and corresponding profile are shown in magenta, the PTV in black symmetrically around the CTV. The blue profile shows the change in dose distribution for the higher density target tissue moved laterally by 5mm (CTV borders in blue, center at -8.0cm). The intersection of the profiles with the borders of the CTV shows, that the CTV is covered by the 95% isodose in both cases.

The resulting change in dose distribution is shown in fig 8. For the accurate dose calculation algorithms the dose distribution changes as the target moves i.e. the location of the higher density tissue inside the lung changes. The steepened dose gradients in the periphery of the CTV thereby kind of "travel with the CTV". This means, that the PTV is not encompassed by the 95% isodose, because the dose is calculated based on lung tissue at the location of the PTV periphery. If the target moves towards the surface of the PTV, the 95% isodose moves there as well and the tumor tissue is still encompassed by the same isodose. Therefore the evaluation of the plan optimization was based on the CTV instead of the PTV.

## Discussion

The goal of the study was to optimize high dose precision radiation therapy of small lung lesions using accurate dose calculation algorithms. In a first step, a non-modulated 9 field conformal treatment plan was created on an inhomogeneous phantom, calculated with Pencil Beam, Collapsed Cone, and Monte Carlo, and validated by 2D film measurements. Then the plan was optimized by adding small segments to the peripheral part of the target in the sense of a two-step intensity modulation in order to increase target coverage and dose homogeneity without compromising the dose to the lung.

### Accuracy of dose calculation

The measurements showed a very good agreement with the Monte Carlo dose calculation, with a maximum difference of up to 3%, which is in within the accuracy of film dosimetry of ± 3%. The Pencil Beam algorithm overestimated the dose compared to the film by up to 15% at the interface of the target to the lung, whereas the Collapsed Cone underestimated the dose in this region by up to -8%.

These results confirm the accuracy of the MC calculations and the overestimation of the dose by the Pencil Beam algorithm as it was published in several studies [5-10]. This can be explained by the fact that PB calculations do not model the re-buildup correctly because electron transport is not taken into account. As well, only a one dimensional longitudinal inhomogeneity correction is performed whereas lateral tissue heterogeneities are neglected.

The underestimation of the dose by the Collapsed Cone algorithm, however, has not been so clearly stated until now. Aspradakis et al [23] reported that the monitor units calculated with CC were within ± 2% of the measurements. For tissue heterogeneities the maximum deviation was 3.8% at the central axis for a large lung block of 25 cm × 25 cm × 10 cm in a large water equivalent block of 50 cm × 50 cm × 20 cm. In the penumbra region, however, a maximum dose deviation of 28% could be found. For missing backscatter the error was up to 5%. Weber and Nilsson [24] reported good agreement of the Collapsed Cone algorithm with measurements in most cases. However, calculations were outside the limits of 5% in the buildup region.

Haedinger et al [10] found an average difference in absolute dose of 5.4% (SD 5.8%) for the Collapsed Cone algorithm compared to the Pencil Beam algorithm, increasing with decreasing sizes of the CTV (2 cm^3 ^to 256 cm^3^). The shape of the MC profiles resembled the Collapsed Cone calculations more closely, however, only relative and no absolute profiles were compared, and no measurements were performed. Koelbl et al [9] performed measurements with a 6MV photon beam in a phantom consisting of slices of styrofoam (lung parenchyma) and RW3 (chest wall). The beam direction was parallel to the tissue interfaces. In this setup, the dose measured with an ion chamber at 15 cm depth in the tumor tissue part of the phantom showed a difference of only 1.2% compared to the CC calculations.

The relatively high differences between the dose calculated by Collapsed Cone and the measured dose found in our study can be explained by the fact that the calculations were performed for 9 fields with perpendicular incidence to the lung/tissue interface of a small lung lesion of 2 cm diameter and 4 cm height. In this case incorrect modeling of the secondary buildup as described by Weber and Nilsson [24] as well as missing backscatter as described by Aspradakis et al [23] have a large influence on the calculated dose.

### Plan optimization

It is possible to improve target coverage by simply renormalizing the 9 field Collapsed Cone plan to the isocenter, thus increasing the number of MU and dose to the ipsilateral lung. Using additional peripheral segments, however, also the dose homogeneity can be improved and the dose to the ipsilatieral lung can be lowered. Lower weighting of the peripheral segments was not possible for a prescription dose of 2Gy because the linac handles only integer MU. For higher prescription doses as used in clinical practice, even lower weighting of the segments might be worth considering.

### Moving Targets

In principle the technique is applicable to moving targets also. In this case the PTV margins have to be increased in the cranio-caudal direction, which is the main direction of the movement of targets in the lung. The field size of the additional segments as well as of the open fields have to be increased in cranio-caudal direction accordingly.

If additional segments are used, the dose gradient gets steeper. If the dose is now prescribed to the same isodose encompassing the PTV, the part of the lung which is outside the PTV will get a lower dose, but the part of the lung inside the PTV will get a higher dose. It is therefore recommended to use breath hold or gating techniques to be able to keep PTV margins as small as possible.

Since the lung density changes with exhalation and inhalation, the dose distribution can be modeled correctly only if the treatment is performed in the same status of exhalation or inhalation as the planning CT [25]. The lowest dose to the lung will be achieved for deep inhalation, since the percentage of irradiated lung volume decreases as the overall lung volume increases.

## Conclusion

The use of forward 2-step intensity modulation in radiotherapy of small lung lesions allows the improvement of tumor coverage and dose homogeneity with a somewhat lower dose to the lung compared to non-modulated treatment plans and may thus help to increase the local tumor control probability. While the Collapsed Cone algorithm agrees better with measurements than the Pencil Beam algorithm, both algorithms have limited accuracy at tissue/lung interfaces. Since this is especially problematic for small targets, Monte-Carlo methods should be used in this situation. Breath hold or gating techniques are recommended in order to keep the PTV margins small.

## List of Abbreviations

2D 2 Dimensional

PB Pencil Beam Algorithm

CC Collapsed Cone Algorithm

CTV Clinical Target Volume

DVH Dose Volume Histogram

MC Monte Carlo

PTV Planning Target Volume

## Competing interests

The author(s) declare that they have no competing interests.

## Authors' contributions

BD participated in the conception and design of the study, carried out the planning study, evaluated the results and drafted the manuscript. CW performed the film measurements in the phantom. AK commissioned the Monte Carlo system. DF participated in the planning process. RL designed and implemented the CT data import for the Monte Carlo system. MP and FS performed the validation of the Gafchromic EBT films. FW participated in the design of the study and revised the manuscript. FL conceived of the study, participated in its design and coordination and helped to draft the manuscript.
